# Impaired Ratio of Unsaturated to Saturated Non-Esterified Fatty Acids in Saliva from Patients with Cystic Fibrosis

**DOI:** 10.3390/diagnostics10110915

**Published:** 2020-11-08

**Authors:** Monica Gelzo, Paola Iacotucci, Vincenzo Carnovale, Alice Castaldo, Marika Comegna, Gustavo Cernera, Gaetano Corso, Giuseppe Castaldo

**Affiliations:** 1 Dipartimento di Medicina Molecolare e Biotecnologie Mediche, Università di Napoli Federico II, 80131 Napoli, Italy; monica.gelzo@unina.it (M.G.); marika.comegna@unina.it (M.C.); gustavo.cernera@unina.it (G.C.); 2 CEINGE-Biotecnologie Avanzate, 80145 Napoli, Italy; alice.castaldo@unina.it; 3 Dipartimento di Scienze Mediche Traslazionali, Università di Napoli Federico II, 80131 Napoli, Italy; paola.iacotucci@unina.it (P.I.); vincenzo.carnovale@unina.it (V.C.); 4 Dipartimento di Sanità Pubblica, Università di Napoli Federico II, 80131 Napoli, Italy; 5 Dipartimento di Medicina Clinica e Sperimentale, Università di Foggia, 71121 Foggia, Italy; gaetano.corso@unifg.it

**Keywords:** cystic fibrosis, saliva, non-esterified fatty acids, inflammation, lung disease

## Abstract

Impaired salivary non-esterified fatty acids (NEFA) levels have been previously observed in cystic fibrosis (CF). This study aimed to characterize the salivary NEFA profile in CF and to examine whether the alterations are related to the pancreatic status and/or lung disease severity. We analyzed salivary NEFA, cholesterol and interleukin-6 (IL-6) in CF patients (*n* = 66) and healthy subjects (*n* = 48). CF patients showed higher salivary levels of cholesterol, total NEFA (that was negatively correlated with serum triglycerides), unsaturated NEFA/saturated NEFA (U/S NEFA) ratio and IL-6 than controls. The U/S NEFA ratio was positively correlated with IL-6 in both patients and controls, suggesting an association between this parameter and local inflammation independently from the disease. No correlation between salivary lipids and pancreatic status was observed, while the U/S NEFA ratio was higher in patients with severe lung disease than mild/moderate severity and may represent a prognostic marker of lung disease in CF.

## 1. Introduction

Cystic fibrosis (CF) is the most common autosomal recessive disorder in Caucasians. CF is due to mutations in the cystic fibrosis transmembrane conductance regulator (*CFTR*) gene, leading to an altered sodium and chloride transport through the respiratory, biliary, gastrointestinal and reproductive tract epithelia [1]. Morbidity and mortality in patients with CF are mainly due to lung disease [2]. In fact, an ex-vivo model of nasal epithelial cells from CF patients has been proposed to study new and personalized therapies [3,4]. The inflammation plays a fundamental role in CF lung disease [5]. Currently, the procedures for monitoring the inflammatory and infectious states are mainly performed on sputum and bronchoalveolar lavage fluid (BALF) [6,7]. Given the difficulty and invasiveness of collecting these biological matrices, actual research is aiming to find new biomarkers of CF lung disease on new biological matrices. Saliva has been proposed as a valid noninvasive alternative to BALF given its association with the respiratory apparatus [8].

Recent studies have shown elevated levels of inflammatory cytokines in CF airway epithelia [9], as well as in sputum, BALF and stimulated saliva [9,10,11], although the causative mechanism of increasing salivary cytokines in CF is poorly known.

Interestingly, it has been observed that the production of inflammatory cytokines in mature macrophages is induced by non-esterified fatty acids (NEFA) [12]. An opposed effect has been observed in other cells by short fatty acids [13,14]. Furthermore, a positive correlation between plasma interleukin-6 (IL-6) and NEFA has been reported [15]. Circulating NEFA derive mainly from the lipolysis of triglycerides by pancreatic enzymes and subsequent absorption by bile salt micellization in a tightly regulated mechanism [16]. About 85% of patients with CF are affected by pancreatic insufficiency (PI), which leads to impaired lipolysis and fat malabsorption [17]. Furthermore, alterations of unsaturated fatty acids have been found in the plasma and tissues of patients with CF, but the hypothesis of an association with fat malabsorption was recently questioned [18,19].

In saliva, NEFA represent the main component among lipids, and their levels depend on serum transudate, exfoliative cells and gland secretion. Alterations of salivary lipid homeostasis have been observed in CF, as well as in other systemic diseases, e.g., diabetes and Sjögren’s syndrome [20]. In particular, a significant increase of total NEFA and cholesterol in stimulated saliva from ten young patients with CF was reported [21], but little is known about the salivary NEFA profile and its association with clinical status, particularly with CF-related pulmonary disease [22].

On the basis of this background, we aimed to examine whether alterations of salivary lipids are associated with pancreatic status and/or lung disease severity. To this purpose, we analyzed salivary free cholesterol, NEFA profile and IL-6 in adult patients with CF and healthy subjects.

## 2. Materials and Methods

### 2.1. Study Population

Adult patients with CF were randomly recruited at the Regional Cystic Fibrosis Reference Center of University of Naples Federico II with the approval of the Ethics committee of Federico II University Hospital (244_2015) from September 2016 to December 2016. The study was performed according to current version of the Helsinki Declaration, and written informed consent was obtained from all patients. All enrolled patients received a confirmed diagnosis of CF, accordingly with the current guidelines [23], and CFTR genotype was determined by screening for a mutations panel and for the most frequent rearrangements [24]. When we did not detect mutations in one or both alleles, *CFTR* gene sequencing was carried out [25]. As a control group, we recruited adult healthy volunteers, which were age and sex-matched to CF patients, from January 2017 to April 2018.

In all CF patients, the pancreatic status was defined at the time of diagnosis by two of these tests: 3-day fecal-fat balance tests, steatocrit measurements and fecal pancreatic elastase 1 (FE-1). In this study, the enrollment of CF patients with PI or with pancreatic sufficiency (PS) was performed on the basis of the last FE-1 value. Patients with FE-1 <200 μg/g of feces were defined as PI-CF and those with FE-1 ≥200 μg/g of feces as PS-CF. All patients with CF were underwent clinical evaluation of the respiratory apparatus at the time of saliva and blood collection. For each patient, we classified CF lung disease as severe, moderate and mild on the basis of age and the forced expiratory volume (FEV1) [26], which was expressed as a predicted value percentage (% pred) [27].

All CF patients followed the Mediterranean diet, with a fat intake depending on pancreatic status. Specifically, the total fat intake for PI-CF patients was, on average, 42% of 2600 Kcal/day, with saturated, monounsaturated and polyunsaturated fatty acid intakes of 11%, 17% and 7% of total fats, respectively. Instead, the total fat intake for PS-CF patients was, on average, 34% of 2300 Kcal/day, with saturated, monounsaturated and polyunsaturated fatty acid intakes of 8%, 16% and 3%, respectively.

### 2.2. Samples

We collected resting saliva samples (i.e., without stimulation) in the morning (9–12 a.m.), after two hours at least of fasting, from all CF patients and controls. Resting saliva (about 1–3 mL) was collected in sterile plastic tubes in ice and then centrifuged for 30 min at 14,000 *g* to remove bacteria/cellular debris. In order to prevent in vitro auto-oxidation processes [28], the supernatants were spiked with butylated hydroxytoluene at the final concentration of 1 mg/mL and stored at −80 °C until analysis. Blood samples were also collected after an overnight fast from patients with CF. Serum was separated from blood cells and analyzed for serum total cholesterol and triglycerides determinations by an automated biochemistry analyzer (Architect ci16200 Integrated System, Abbott Diagnostics, Rome, Italy), as previously described [29].

### 2.3. Salivary Non-Esterified Lipid Analysis

Analytical high-performance liquid chromatography solvents, including methanol, ethanol, chloroform, hexane and dichloromethane, were obtained from JT Baker (Deventer, Netherlands). Chloridric acid (37%; Sigma, St Louis, MO, USA) was diluted at a final concentration of 1N. The pentadecanoic acid (C15:0; Sigma, St. Louis, MO, USA) was dissolved in ethanol at a final concentration of 0.2 mg/mL. Boron trifluoride/methanol (10%, *w*/*w*) was purchased from Supelco (Bellefonte, PA, USA). 5-α-cholestane (Sigma, St Louis, MO, USA) was prepared as a 0.4-mg/mL stock solution. Salivary non-esterified fatty acids (NEFA) and free cholesterol were extracted from saliva as previously described [30] and slightly modified. Briefly, for the simultaneous analysis of free cholesterol and NEFA, saliva (1 mL) was spiked with 5α-cholestane (10 μg) and C15:0 (10 μg) used as internal standards for the quantitative analysis of free cholesterol and NEFA, respectively. Then, the sample was acidified with chloridric acid (100 μL), mixed vigorously with saline solution (3 mL) and incubated in ice for 5 min. Non-esterified lipids were extracted two times with chloroform (5 mL). The lower layers (lipophilic phase) were washed with 0.45% sodium chloride solution and dried under a nitrogen stream. The dried residue was resuspended with boron trifluoride/methanol (500 μL) and incubated at 60 °C for 20 min. The solution was mixed with distilled water (1 mL), and methylated lipids were extracted with hexane (1 mL × 2). Finally, the sample was dried under nitrogen, and the residue was dissolved in 100 μL of dichloromethane. Qualitative and quantitative analyses of the methylated lipids were performed, respectively, by gas chromatography coupled with mass spectrometry (GC-MS) and GC coupled to a flame ionization detector (GC-FID), as previously described [31]. The mean of the coefficient of variation, calculated for each lipid from all sample duplicates, was less than 10.7% (standard deviation, SD: 3.7%).

### 2.4. Salivary IL-6 Analysis

The measurement of the salivary IL-6 levels was performed by an enzyme-linked immunosorbent assay using a human IL-6 ELISA Max™ Set Deluxe kit (BioLegend, Inc., San Diego, CA, USA), in accordance with the manufacturer’s instructions. All saliva samples and standard solutions for calibration curves were analyzed in duplicate using 100 μL of the sample. The concentration values of IL-6 were expressed as pg/mL.

### 2.5. Statistical Analysis

Normality of data distributions was assessed by the Shapiro-Wilk test. Data with normal distributions were reported as mean (SD), while nonparametric distributions were reported as median (interquartile range, IQR). Categorical data were reported as frequency (percentage), and the chi-square test was used to compare the frequencies. The comparisons between two groups with nonparametric distributions were performed by the Mann-Whitney U test. Statistical differences between three groups with normal distributions were assessed by ANOVA and Bonferroni’s correction as a post-hoc test. The correlations between variables with nonparametric distributions were evaluated by Spearman’s rank-order correlation, and Spearman’s rank correlation coefficient (r_s_) was calculated. All probabilities were two-tailed, and *p*-values <0.05 were considered as significant.

## 3. Results

### 3.1. Salivary Lipid Composition and IL-6 Levels in Healthy Subjects and Patients with CF

Table 1 shows the demographic and clinical characteristics of patients with CF and healthy subjects (controls). No significant differences of demographic data were observed between the two groups.

The levels of non-esterified lipids, i.e., free cholesterol and NEFA, in resting saliva samples from controls and adult patients with CF are reported in Table 2. Salivary free cholesterol results were significantly higher in CF patients than in the controls. Among NEFA, we found measurable levels of palmitic (C16:0), stearic (C18:0), oleic (cis-C18:1) and linoleic (C18:2) acids (Table 2). Other NEFAs, such as lauric (C12:0), myristic (C14:0), palmitoleic (C16:1), margaric (C17:0) and elaidic (trans-C18:1) acids, were also detected, but their levels were lower than the limit of quantification of the method (0.01 mg/L).

The total NEFA levels were significantly increased in CF patients compared to controls. The concentrations of all measured NEFA increased overall in CF patients compared to the controls, but the percentages of increase (Δ, %) of unsaturated NEFA, i.e., cis-C18:1 and C18:2, were much higher than those of saturated NEFAs, i.e., C16:0 and C18:0 (Table 2). Therefore, we calculated the salivary unsaturated/saturated NEFA ratio (U/S NEFA ratio, %) as follows:

U/S NEFA ratio = [(cis-C18:1 + C18:2) / (C16:0 + C18:0)] × 100(1)

**Figure 1:**
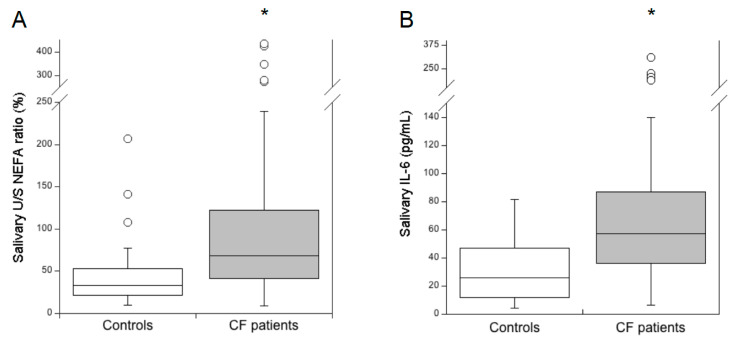
Comparison of the salivary unsaturated/saturated NEFA ratio (U/S NEFA ratio) (**A**) and interleukin 6 (IL-6) (**B**) between healthy subjects (controls, *n* = 48) and cystic fibrosis (CF) patients (*n* = 66). * *p* < 1 × 10^-6^, Mann-Whitney U test. The U/S NEFA ratio was calculated by Equation (1).

The U/S NEFA ratio results were significantly higher in CF patients than the controls (Figure 1A; *p* < 1 × 10^-6^). Salivary IL-6 levels were also significantly higher in CF patients than controls (Figure 1B; *p* < 1 × 10^-6^). We evaluated the correlations between the salivary U/S NEFA ratio and salivary IL-6 levels in patients with CF and in the controls. The Spearman’s rank-order correlation analysis showed a significant positive correlation between IL-6 levels and the U/S NEFA ratio in CF patients (r_s_ = 0.365, *p* = 0.001), as well as in the controls (r_s_ = 0.428, *p* = 0.005).

### 3.2. Salivary Lipids versus Serum Lipids and Pancreatic Status in CF Patients

**Figure 2:**
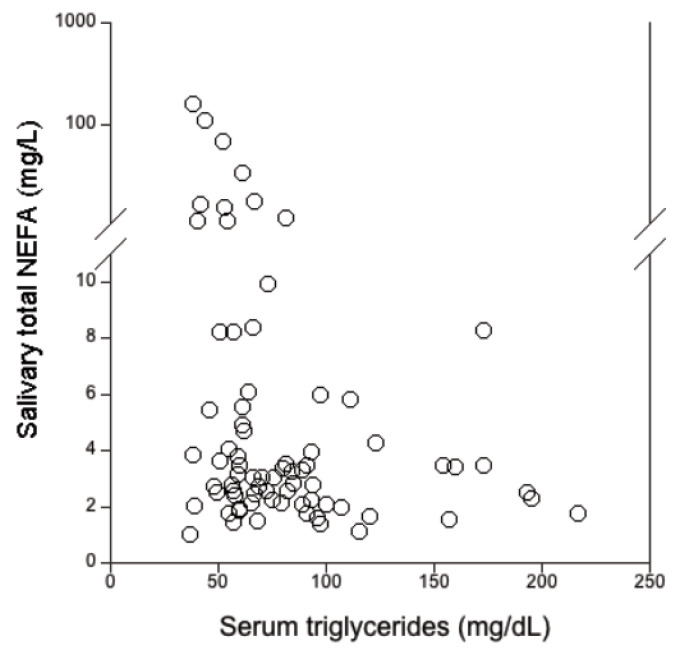
Correlation between the levels of serum triglycerides and salivary total NEFA in patients with CF (*n* = 66). Spearman’s rank -order correlation: r_s_ = -0.281, *p* = 0.014.

We found that the salivary free cholesterol levels in CF patients did not correlate with the serum total cholesterol that was equal to 150 (122–180) mg/dL. On the other hand, the salivary total NEFA were negatively correlated with the serum triglycerides (Figure 2; r_s_ = -0.281, *p* = 0.014) that resulted equal to 82 (60–109) mg/dL.

We did not find significant differences between the salivary lipid compositions of CF patients with pancreatic sufficiency (PS-CF, *n* = 29) and pancreatic insufficiency (PI-CF, *n* = 37) (Supplementary Table S1).

Although the fat intake from the diet was higher in PI-CF patients (120 g/day) than PS-CF patients (90 g/day), the ratio of total unsaturated/total saturated fatty acids from the diet were comparable and equal to 2.18 and 2.38 in PI-CF and PS-CF patients, respectively.

### 3.3. Salivary Lipids versus Lung Disease Severity

**Figure 3:**
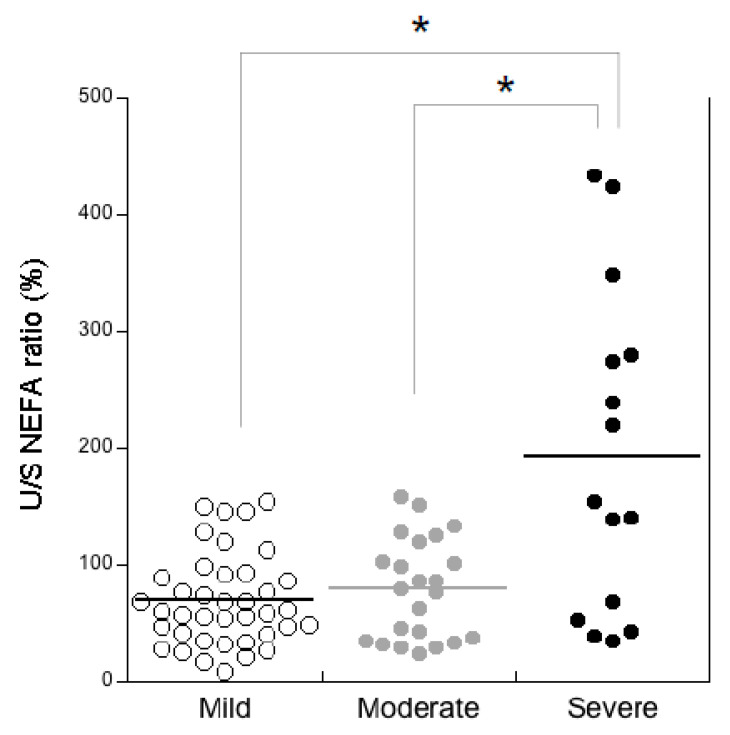
Comparison of the salivary unsaturated/saturated NEFA ratio (U/S NEFA ratio) among CF patients with mild (*n* = 31), moderate (*n* = 20) and severe (*n* = 15) lung disease. * *p* < 0.0001; severe versus mild and moderate, ANOVA.

We compared the salivary lipids among three severity levels of CF-related lung disease, i.e., mild (*n* = 31), moderate (*n* = 20) and severe (*n* = 15). Significant differences were found for the U/S NEFA ratio (Figure 3) that was significantly higher in CF patients with severe lung disease than those with mild and moderate severity (*p* < 0.0001).

No significant difference was observed between mild and moderate severity. However, the U/S NEFA ratio in both the mild (median: 59.2%; IQR: 40.5–91.5%) and moderate (median: 79.5%; IQR: 34.7–119.6%) groups were significantly higher (*p* = 0.0006, Mann-Whitney U test) than the controls (median: 33.3%; IQR: 21.3–53.6%).

No significant differences of the ratio of total unsaturated/total saturated fatty acid intakes from the diet were observed among the three patient groups.

## 4. Discussion

This study describes the salivary lipids composition, i.e., free cholesterol and NEFA profile, in adult patients with CF and healthy subjects. We found higher salivary cholesterol levels in CF patients compared to controls, accordingly with the literature [20]. However, we found no correlation between salivary and serum cholesterol levels in CF patients. In fact, we previously reported a significant reduction of plasma cholesterol in patients with CF compared to healthy subjects [32]. The increased free cholesterol in saliva from CF patients may be due to an accumulation of free cholesterol in subcellular compartments of the salivary gland, as demonstrated in lung and trachea tissues from CF animal models [33], but further studies are need to investigate regarding the true mechanism.

Total salivary NEFA results were significantly increased in CF patients compared to the controls, in agreement with literature [21]. Furthermore, a significant negative correlation between the total salivary NEFA and serum triglycerides was found in CF patients. NEFA are produced by triglyceride lipolysis in response to an energy demand from the organism [16], and an increased expenditure of energy associated to fat malabsorption is typical of patients with CF [34]. Hence, the increase of NEFA in saliva could mirror a potential increase of circulating NEFA, as previously reported in the literature [35]. Herein, plasma NEFA were not measured, and this lack represents a study limitation.

The salivary NEFA profile observed in our controls agrees with that reported by Kulkarni et al. [36] in healthy subjects. In CF patients, we found significantly higher concentrations of salivary unsaturated NEFA and saturated NEFA compared to the controls. The increase of cis-C18:1 levels could be due to an increased expression of Δ9-desaturase that was previously reported in CF cultured cells [37]. In addition, some studies in CF epithelial cells showed that the increased expression of fatty acids desaturases is caused by enhanced AMP-activated protein kinase activity due to the lack of CFTR activity [38,39]. Moreover, some previous studies suggested that the increased activity of desaturases may cause the increased levels of prostaglandins and leukotrienes in the airways of patients with CF [40,41], which correlates with the severity of CF-related pulmonary disease [41,42].

Furthermore, we found increased levels of C18:2 that represent an essential fatty acid, as well as the precursor of prostaglandins and leukotrienes via the arachidonic acid pathway. In contrast with our results, a previous literature report decreased the C18:2 levels in plasma, tissues and cells from CF patients [18,19]. However, in these studies, the authors measured the total fatty acids, i.e., the sum of NEFA and esterified fatty acids, while, in our study, we determined only the NEFA that represented the primary component of salivary lipids [20]. Bhura-Bandali et al. [43] showed that the ΔF508 mutation in the CFTR gene causes a reduced incorporation of C18:2 in the membrane phospholipids of epithelial cells. Hence, the increased salivary levels of free C18:2 observed in our CF patients may be caused by a reduced uptake of this fatty acid in phospholipid biosynthesis in epithelial cells of the salivary gland [44,45].

The salivary IL-6 levels were significantly higher in CF patients than in the controls. These findings agree with our previous study, wherein we found higher salivary IL-6 levels in CF patients with inferior turbinate hypertrophy than CF patients without sinonasal complications [46]. Herein, we found also an impaired U/S NEFA ratio due mainly to the higher increase percentage of unsaturated NEFA, i.e., cis-C18:1 and C18:2, than saturated NEFA. This ratio’s results were two-fold higher in CF patients than the controls. We did not find correlations between the U/S NEFA ratio and pancreatic status. On the other hand, the salivary U/S NEFA ratio was positively related to salivary IL-6 in both the CF patients and controls, suggesting an association between the U/S NEFA ratio and local inflammation independently from the disease. Interestingly, we found significantly higher values of the U/S NEFA ratio in at least 50% of CF patients with severe lung disease than patients with mild and moderate severity. We suggest that this salivary parameter may help to distinguish patients with severe lung disease.

A study limitation was represented by an unmatched diet for the controls. However, no significant differences were observed in the intakes of saturated/unsaturated fatty acids among the CF patients.

## 5. Conclusions

Herein, we described abnormalities of salivary lipids in patients with CF. In particular, we observed a significant increase of the U/S NEFA ratio that was associated with salivary IL-6 levels, as well with lung disease severity in CF patients. Overall, our results, together with the findings of previous studies, suggested that inflammation may drive lipid abnormalities in CF. Although preliminary, our findings suggested that the salivary U/S NEFA ratio may represent a potential prognostic marker for monitoring lung disease in these patients. Further investigations on saliva samples from a higher number of patients with CF are needed to validate these results.

## Figures and Tables

**Table 1:** Demographic and clinical data of the study population.

	Controls (*n* = 48)	CF Patients (*n* = 66)
Age (years)	27 (23–37)	23 (18–39)
Males, *n* (%)	26 (54.2)	37 (56.1)
Lung disease severity, *n* (%):		
severe	-	15 (22.7)
moderate	-	20 (30.3)
mild	-	31 (47.0)
Pancreatic insufficiency, *n* (%)	-	37 (56.1)

Nonparametric data are reported as the median (IQR), and the comparisons were performed by the Mann Whitney U test. Categorical data are reported as the frequency (percentage), and the chi-square test was used to compare the frequencies. CF: cystic fibrosis.

**Table 2:** Comparison of salivary free cholesterol and non-esterified fatty acid (NEFA) levels in controls and adult patients with CF.

Salivary Lipids (mg/L)	Controls (*n* = 48)	CF Patients (*n* = 66)	Δ (%) ^a^	*p*-Value ^b^
Free cholesterol	0.38 (0.22–0.65)	0.58 (0.36–1.09)	+52.6	**0.01**
Total NEFA	2.26 (1.20–3.70)	3.29 (2.24–5.49)	+45.6	**0.001**
C16:0	0.78 (0.38–1.10)	0.95 (0.69–1.55)	+21.8	**0.01**
C18:0	0.67 (0.32–1.30)	0.82 (0.58–1.26)	+22.4	0.139
cis-C18:1	0.35 (0.17–0.62)	0.75 (0.49–1.34)	+114.3	**3.5 × 10^−7^**
C18:2	0.16 (0.08–0.32)	0.42 (0.25–0.95)	+162.5	**2.3 × 10^−7^**

Nonparametric data are reported as the median (IQR), and the comparisons were performed by the Mann Whitney U test. ^a^ Differences between the median values in the controls and CF patients, expressed as percentages. ^b^ Values in bold indicate significant differences.

## Supplementary Materials

The following are available online at https://www.mdpi.com/2075-4418/10/11/915/s1: Table S1. A comparison of salivary free cholesterol and non-esterified fatty acids (NEFA) levels in CF patients with pancreatic sufficiency (PS-CF) and insufficiency (PI-CF).
