# Supplementary material for: Impaired Ratio of Unsaturated to Saturated Non-Esterified Fatty Acids in Saliva from Patients with Cystic Fibrosis
Source: Diagnostics (Basel). 2020 Nov 8;10(11):915. doi: 10.3390/diagnostics10110915 (PMC7695280; doi:10.3390/diagnostics10110915)
Supplement: Supplementary file 1 [file diagnostics-10-00915-s001.pdf]

diagnostics

Supplementary Materials

Table S1. Comparison of salivary free cholesterol and non-esterified fatty acids (NEFA) levels in CF patients with pancreatic sufficiency (PS-CF) and insufficiency (PI-CF).

Lipids (mg/L)	PS-CF (n = 29)	PI-CF (n = 37)
Free cholesterol	0.83 (0.35–1.79)	0.52 (0.41–1.12)
Total NEFA	3.11 (2.09–4.05)	2.80 (1.99–3.75)
C16:0	0.93 (0.66–1.21)	0.90 (0.70–1.39)
C18:0	0.86 (0.59–1.12)	0.82 (0.60–1.08)
cis-C18:1	0.62 (0.41–0.91)	0.53 (0.33–0.94)
C18:2	0.31 (0.19–0.55)	0.26 (0.18–0.42)
U/S NEFA ratio (%)	58.5 (31.4–86.2)	46.3 (34.8–56.3)

Data with non-parametric distributions are reported as median (interquartile range) and the comparisons have been performed by Mann Whitney *U* test. U/S NEFA ratio was calculated as follows: [(*cis*-C18:1 + C18:2) / (C16:0 + C18:0)] × 100.
